# A Good Disinfectant

**Published:** 1885-03

**Authors:** 


### A GOOD DISINFECTANT.

The following compound has been presented to the Berlin Medical Society for purifying the atmosphere of the sick room:

Oils of rosemary, lavender and thyme, in the proportions of 10, 2½, and 2½ parts, respectively, are mixed with water and nitric acid in the proportion of 30 to 1½. The bottle should be shaken before using, and sponge saturated in the compound and left to diffuse by evaporation. This compound is said to possess extraordinary properties in controlling odors and effluvia.

